# Clinical usefulness of next-generation sequencing-based target gene sequencing in diagnosis of inherited bone marrow failure syndrome

**DOI:** 10.1007/s00277-025-06392-0

**Published:** 2025-05-13

**Authors:** Young Dai Kwon, Kyung Taek Hong, Juyeon Lee, Yoon Sunwoo, Yeseul Kim, Sung Im Cho, Hyun Jin Park, Bo Kyung Kim, Jee-Soo Lee, Jung Yoon Choi, Moon-Woo Seong, Hyoung Jin Kang

**Affiliations:** 1 Department of Pediatrics, Seoul National University College of Medicine, Seoul, Korea, 101, Daehak-ro, Jongno-gu, Seoul, 03080 Republic of Korea; 2 Seoul National University Cancer Research Institute, Seoul, Republic of Korea; 3 Department of Laboratory Medicine, Seoul National University Hospital, Seoul National University College of Medicine, Seoul, Republic of Korea; 4 Wide River Institute of Immunology, Hongcheon, Republic of Korea

**Keywords:** Next-generation sequencing, Inherited bone marrow failure syndrome, Cytopenia

## Abstract

Inherited bone marrow failure syndromes are genetic hematologic disorders with increased cancer risk. Accurate diagnosis is crucial for appropriate management. This study assessed the clinical usefulness of next-generation sequencing (NGS)-based target gene sequencing in pediatric and AYA (adolescent and young adult) patients with hematologic abnormalities. From December 2019 to June 2023, 93 patients with suspected congenital hematologic diseases at a single institution underwent NGS-based testing. Medical records were retrospectively reviewed. The median age at diagnosis was 9.3 years (range 0.2–31.4), with 59.1% males. Indications for testing included specific medical histories (28 patients), persistent cytopenia or recurrent neutropenic fever (22 patients), changes in cytopenia patterns (11 patients), and other reasons (32 patients). Pathogenic variants were identified in 9/28 (32.1%), 3/22 (13.6%), 4/11 (36.4%), and 0/32 (0%). Overall, 16 patients (17.2%) had pathogenic variants, including *FANCA*, *BRCA2*, *PMS2*, *ELANE*, *G6PC3* and *VPS13B* in patients with idiopathic neutropenia, and *GATA2* in patients with suspected myelodysplastic syndrome. Genetic findings led to diagnostic revisions in 12 patients (12.9%), including reclassification of aplastic anemia (AA) as Fanconi anemia, Diamond-Blackfan anemia, or Shwachman-Diamond syndrome, prompting hematopoietic stem cell transplantation and altering cancer surveillance. Pathogenic variants were more frequently observed in patients with a specific medical history or changes in cytopenia, and in those with additional clinical features (cytogenetic abnormalities or non-severe AA). This study demonstrated the diagnostic usefulness of NGS-based target gene sequencing for pediatric and AYA patients with suspected genetic hematologic disorders, supporting the need for multicenter studies and standardized guideline development.

**Supplementary Information:**

The online version contains supplementary material available at 10.1007/s00277-025-06392-0.

## Introduction

Peripheral blood cytopenia can occur in various congenital or acquired disorders in children, adolescents, and young adults. Conditions affecting a single hematopoietic lineage often lack distinctive bone marrow findings. When cytopenia involves two or more lineages, the differential diagnosis includes acquired aplastic anemia (AA), myelodysplastic syndrome (MDS), and inherited bone marrow failure syndromes (IBMFS), as well as immune disorders including hemophagocytic lymphohistiocytosis (HLH) [1]. IBMFS are primarily driven by germline mutations that impair hematopoietic stem cells (HSCs) or hematopoietic stem and progenitor cells (HSPCs), leading to progressive cytopenia due to ineffective hematopoiesis. These mutations lead to over 20 disease entities, including Fanconi anemia (FA), dyskeratosis congenita (DC), Diamond-Blackfan anemia (DBA), Shwachman-Diamond syndrome (SDS), congenital amegakaryocytic thrombocytopenia, and severe congenital neutropenia [2, 3]. IBMFS are genetically heterogeneous and may present with both hematologic and non-hematologic features, including congenital malformations and systemic complications [4–8]. While some patients exhibit distinct morphological traits, the wide variability in disease penetrance and phenotype often leads to overlapping clinical and laboratory features across different IBMFS and related conditions [3, 9–11]. Additionally, patients with IBMFS may not initially present with features of AA; instead, they may first exhibit characteristic physical anomalies, or present signs and symptoms associated with MDS, acute myeloid leukemia (AML), or other conditions. Consequently, diagnosing IBMFS is challenging [12].

Furthermore, accurate genetic diagnosis of IBMFS is crucial due to their association with cancer predispositions such as MDS, AML, squamous cell carcinoma, and sarcoma [4, 6–8]. Precise genetic diagnosis enables appropriate treatment planification, including medication management, HSC transplantation (HSCT), selection of stem cell donors, risk estimation for future complications, and genetic counseling for patients and their families. Moreover, differentiating between acquired and inherited forms of IBMFS is crucial. For instance, the use of immunosuppressive therapy in patients with IBMFS could potentially be harmful [6–8, 12–14]. Traditionally, diagnostic assessments for IBMFS were relied on evaluating family history and physical anomalies, bone marrow examination, and chromosomal breakage testing. However, up to 40% of patients had no significant family history or physical anomalies, representing diagnostic challenges [4]. In addition, conventional genetic methods such as karyotyping, fluorescence in situ hybridization, and Sanger sequencing are limited by low throughput and often require a high index of clinical suspicion. The large number of genes implicated in IBMFS further complicates diagnosis, making gene-by-gene testing both time-consuming and costly.

Over the past decade, the advent of next-generation sequencing (NGS) technology has driven substantial progress in genetic diagnostics and was quickly integrated into clinical practice. NGS, encompassing target gene sequencing, whole-exome sequencing (WES), and whole-genome sequencing (WGS), has emerged as the primary tool for diagnosing inherited diseases, enabling timely and targeted treatment. By allowing parallel analysis of multiple disease-associated genes, NGS addresses the limitations of conventional methods, which are restricted by low throughput and diagnostic inefficiency [3–6, 15–22]. While various genetic causes and mechanisms underlying IBMFS are well-established [1, 10, 15], diagnostic uncertainty remains a significant challenge.

In this clinical context, the significance of NGS-based target gene sequencing is increasingly acknowledged, as it enables the comprehensive and rapid identification of causative genes, leading to accurate diagnoses in patients with suspected hereditary conditions. Notably, previous studies have demonstrated that NGS can achieve a diagnostic yield of up to 50% in patients with suspected IBMFS. [3, 5, 18, 23–26].

This study aimed to investigate the clinical usefulness of NGS-based target gene sequencing in pediatric and AYA (adolescent and young adult) patients presenting with hematological abnormalities and associated symptoms, and to propose suitable indications for NGS-based target gene sequencing based on hematological or clinical findings. Although IBMFS can be diagnosed at any age, this study focuses on patients from a single pediatric institution to highlight the importance of early genetic diagnosis, which facilitates timely therapeutic decisions and long-term care planning in this population.

## Materials and methods

### Patients and data collection

From December 1, 2019, to June 30, 2023, a total of 93 consecutive patients who were clinically suspected of having congenital hematologic diseases at Seoul National University Children's Hospital were included in this study. None of the patients had previously undergone genetic tests.

This study was designed as a retrospective analysis of medical records, including clinical information, laboratory results, and other relevant data. The collected data included demographics, past medical and family history, associated symptoms, supplementary diagnostic tests (such as bone marrow examination, cytogenetics, pathogenic virus testing), and therapeutic interventions (such as medication and HSCT).

This study was approved by the Institutional Review Board (IRB) of the Seoul National University Hospital (IRB number: 2306–177–1443). As this study involved only a review of medical records and no additional interventions for patients, the requirement for written consent was waived.

### Indications of target gene sequencing

**Fig. 1:**
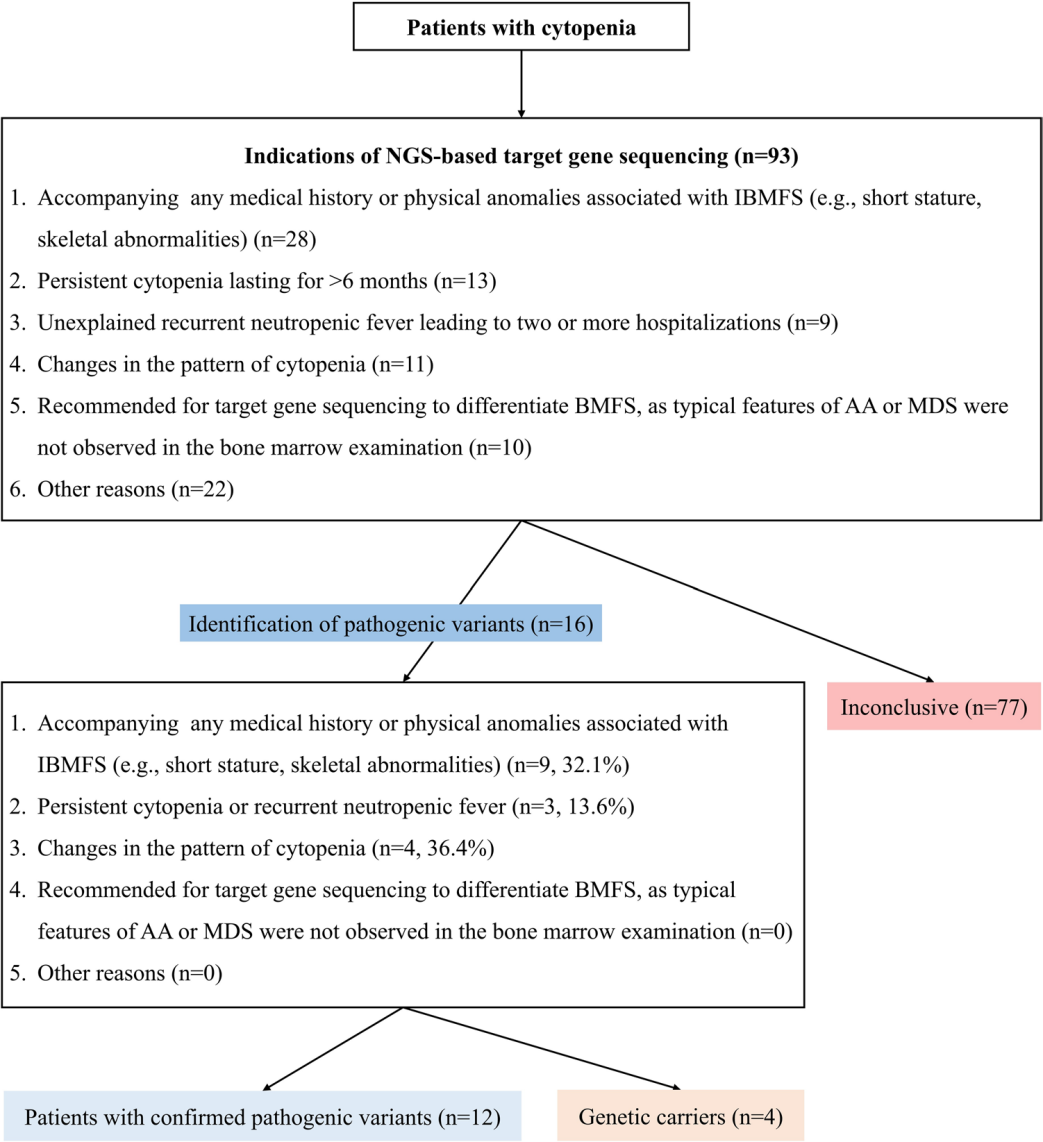
Patient selection and testing outcomes in suspected Inherited bone marrow failure syndromes. NGS = next-generation sequencing, IBMFS = inherited bone marrow failure syndromes, AA = aplastic anemia, MDS = myelodysplastic syndromes

Patients referred to our institution for abnormalities such as persistent cytopenia or physical anomalies underwent NGS-based target gene sequencing if they met any of the following criteria (Fig. 1):

1. Accompanying any medical history or physical anomalies associated with IBMFS (e.g., short stature, skeletal abnormalities)
2. Persistent cytopenia lasting for > 6 months
3. Unexplained recurrent neutropenic fever leading to two or more hospitalizations
4. Changes in the pattern of cytopenia: 1) progression from single lineage to multilineage or 2) aggravating with increased transfusion needs or 3) prolonged aplasia during chemotherapy for malignancies
5. Recommended for target gene sequencing to differentiate bone marrow failure (BMF), as typical features of AA or MDS were not observed in the bone marrow examination

### NGS-based target gene sequencing

We developed the congenital hematologic disease gene panel, an NGS-based assay targeting 96 genes related to IBMFS, including those associated with FA, DC, DBA, SDS, hereditary thrombocytopenia, congenital neutropenia, and other disorders such as GATA2 deficiency and SAMD9/SAMD9L syndrome (See Supplemental Data Table S1). It was designed by our department of laboratory medicine and has been in clinical use since December 2019. DNA was extracted from peripheral blood or bone marrow aspiration in all patients. The SureSelect XT HS2 DNA Reagent Kit (Agilent Technologies, Santa Clara, CA, USA) was used for DNA library preparation and capture according to the manufacturer’s instructions. Targeted fragments were amplified, indexed, and sequenced on the Illumina NovaSeq 6000Dx (Illumina, Inc., San Diego, CA, USA) platform. Until October 2021, bioinformatics processing, from alignment to annotation, was performed with NextGene (Version 2.4.0.1; Software Genetics, State College, PA, USA) [27]. Since then, raw sequence data have been generated as paired-end reads, quality-checked with FastQC, and aligned to the human reference genome using the Burrows-Wheeler Aligner algorithm. Post-alignment, variant calling was performed using GATK HaplotypeCaller, one of the most accurate tools currently available, to identify small variants such as single nucleotide variants and small insertions or deletions. The detected variants were subsequently annotated using snpEff, a software tool that predicts the functional consequences of genetic variants and assists in evaluating their clinical significance. For the comprehensive panel analysis, only variants with a mapping quality score of 20 or higher were filtered to minimize interference from pseudogenes, such as *SBDS*. In addition, Sanger sequencing is routinely conducted alongside NGS for genes known to harbor pseudogenes, and additionally for clinically suspicious patients.

All variants were interpreted and classified based on the American College of Medical Genetics (ACMG) and Genomics and the Association for Molecular Pathology [28]. Only variants determined to be pathogenic or likely pathogenic were recognized as causal mutations. Genetic diagnoses were made based on variant classification in conjunction with the mode of inheritance and relevant clinical findings. Classification was based on a multifactorial evidence framework incorporating allele frequency, predicted functional impact, segregation data, and supporting literature or database entries (e.g., ClinVar, OMIM, HGMD). Common benign variants were filtered using population databases such as the Genome Aggregation Database, Exome Aggregation Consortium, Korean Reference Genome, and Korean Variant Archive, with thresholds adapted to disease prevalence and mode of inheritance. Functional impact was assessed using multiple in silico prediction tools, including Rare Exome Variant Ensemble Learner and Combined Annotation-Dependent Depletion scores to aid the interpretation of missense and splice region variants. Variants of uncertain significance (VUS) were not considered diagnostic but were documented and subjected to further evaluation. All final classifications were confirmed by expert manual review, supplemented by automated interpretation platforms, such as MedyPatho [29].

### Definitions

AA is defined by the coexistence of pancytopenia with unexplained reduced marrow hematopoietic cellularity, and severity is based on peripheral blood counts. Severe aplastic anemia (SAA) is diagnosed when at least two of the following criteria are met: (1) reticulocytes < 60 × 10⁹/L (automated count) or < 20 × 10⁹/L (manual count), (2) platelets < 20 × 10⁹/L, and (3) neutrophil count < 0.5 × 10⁹/L. Very severe aplastic anemia (VSAA) is defined by the same criteria as SAA, with a neutrophil count < 0.2 × 10⁹/L. Non-severe AA (NSAA) refers to a mild to moderate degree of cytopenia that does not fulfill the criteria for SAA or VSAA [30, 31].

### Statistical analysis

To compare the groups with and without confirmed pathogenic variants, as well as the NSAA and SAA subgroups, we used two-sided t-tests for continuous variables and χ2 tests or Fisher's exact tests for categorical variables. All analyses were performed using the R 4.3.2 (The R Foundation for Statistical Computing, Vienna, Austria). P-values of < 0.05 were considered statistically significant. Given the relatively small sample size and the number of variables tested, we applied multiple testing corrections using both the Bonferroni method and the Benjamini–Hochberg false discovery rate approach.

## Results

### Clinical characteristics of the study population

**Fig. 2:**
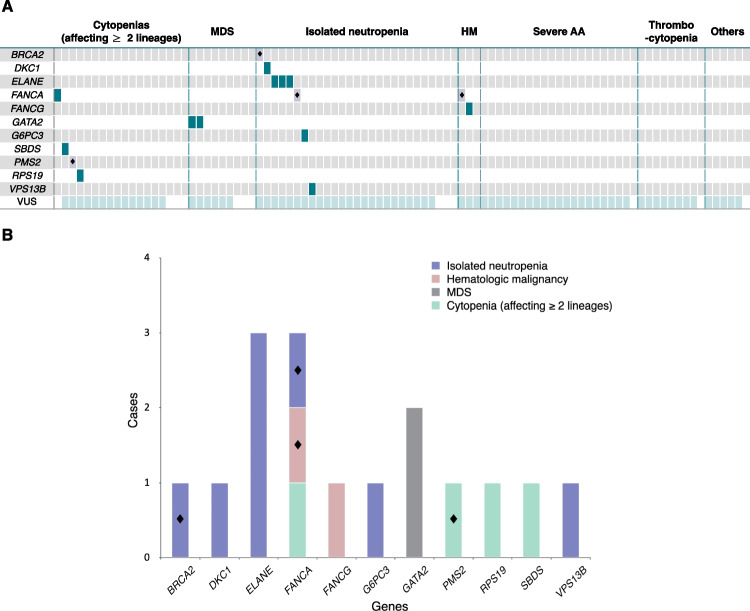
Distribution of pathogenic variants and variants of unknown significance (VUS) in 93 patients. **A** The horizontal axis category represents presumed diagnoses of the patients at the time of next-generation sequencing-based target gene sequencing. Non-severe AA is categorized within the cytopenia (affecting ≥ 2 lineages) group. HM includes acute lymphoblastic leukemia and acute myeloid leukemia, while Others include hemophagocytic lymphohistiocytosis, Langerhans cell histiocytosis, and other conditions such as isolated anemia. Dark green indicates pathogenic variants, black diamonds (◆) represent carriers of pathogenic variants, and light mint green denotes cases with variants of uncertain significance (VUS) (**B**) Frequency and patterns of 16 pathogenic variants identified in 93 patients. MDS = myelodysplastic syndromes, HM = hematologic malignancy, AA = aplastic anemia

Among the 93 patients included in the study, 55 (59.1%) were male, with a mean age of 9.3 years (range 0.2–31.4). Bone marrow and chromosome examinations were performed in approximately 69.9% (65/93) of the patients, among whom 76.9% (50/65) had abnormal bone marrow findings, such as hypocellular marrow for age with or without decreased trilineage hematopoiesis, maturation arrest at the promyelocytic stage, granulocytic hyperplasia, or dyserythropoiesis. Additionally, 9.2% (6/65) had abnormal cytogenetic findings. Chromosome breakage tests were performed on 39.8% (37/93) of patients, revealing positive results in 5.4% (2/37) of patients. Target gene sequencing was used as the first-line diagnostic tool in approximately 46.2% (43/93) of the patients (Table 1). At the time of target gene sequencing, the presumed diagnosis of the patients included isolated neutropenia, cytopenia affecting ≥ 2 lineages including NSAA, SAA, isolated thrombocytopenia, MDS, acute lymphoblastic leukemia (ALL), AML, HLH, and Langerhans cell histiocytosis. The distribution was as follows: 29.0% (27/93), 19.4% (18/93), 22.6% (21/93), 9.7% (9/93), 9.7% (9/93), 2.2% (2/93), 1.1% (1/93), 2.2% (2/93), and 1.1% (1/93) (Fig. 2).

**Table 1:** Clinical characteristics of the study population (*N* = 93)

Characteristic	Values
Age (years)	9.33 (0.17–31.42)
Male sex	55 (59.1)
Hemoglobin (g/dL)	10.5 (6.1–17.7)
White blood cell (/μl)	3120 (900–10850)
Platelet (× 10^3^/μl)	60 (10–613)
Absolute neutrophil count (/μl)	675 (0–4394)
Lowest^a^ Hemoglobin (g/dL)	9.1 (4.5–16.7)
Lowest^a^ White blood cell (/μl)	2780 (90–11400)
Lowest^a^ Platelet (× 10^3^/μl)	38 (1–462)
Lowest^a^ Absolute neutrophil count (/μl)	443 (0–3591)
Patients with positive antinuclear antibody	15/41 (36.6)
Patients with accompanying symptoms (Infection or Bleeding)	61 (65.6)
Patients with abnormal bone marrow findings	50/65 (76.9)
Patients with abnormal cytogenetics findings^b^	6/65 (9.2)
Patients with abnormal chromosomal breakage analysis results	2/37 (5.4)
Patients with family history of hematologic disorders	0 (0.0)
Patients with physical anomalies^c^	12 (12.9)
Patients with medical history^d^	19 (20.4)
Indications of target gene sequencing	
Accompanying any medical history or physical anomalies associated with IBMFS (e.g., short stature, skeletal abnormalities)	28 (30.1)
Persistent cytopenia lasting for more than 6 months	13 (14.0)
Unexplained recurrent neutropenic fever leading to two or more hospitalizations	9 (9.7)
Changes in the pattern of cytopenia	11 (11.8)
Recommended for target gene sequencing to differentiate BMFS, as typical features of AA or MDS were not observed in the bone marrow examination	10 (10.8)
Other reasons (See Supplemental Data Table S2)	22 (23.7)
Patients using target gene sequencing as the first-line diagnostic tool	43 (46.2)
Patients with reported pathogenic variants	16 (17.2)
Patients with confirmed pathogenic variants	12 (12.9)

Values are presented as median (range) or frequency or number (%) or number/total number (%)

^a^ Lowest was defined as the lowest value among results within one month before and after the Gene panel

^b^ Abnormal cytogenetics findings:

45,XX,−7[18]/46,XX[2]; loss of 7

46,XY,del(6)(p22),del(13)(q12q14)[20]

48,XY, + 6, + 8[8]/46,XY[17]

46,XY,−6, + mar[8]/46,XY[5]

61 < 3n >,XXY,−1,−3,−6,−7,−13, + 14,−15,−15,−16,−17,−19,−20, + 21, + mar[6]/46,XY[22]

47,XX, + 1,der(1;7)(q10;p10), + 8[17]/48,idem, + 8[4]

^c^ Physical anomalies: Short stature, Skeletal anomaly, Albinism, Rathke's cleft cyst, Functional single ventricle, Interruption of the aortic arch, Pulmonary vein stenosis, Renal duplication, Congenital esotropia, Atrial septal defect

^d^ Medical history: Graves disease, Type 1 diabetes mellitus with Diabetic Ketoacidosis, Partial Growth hormone deficiency, Neonatal alloimmune thrombocytopenia, Crohn’s disease, Gilbert syndrome, Hepaititis(Autoimmune, Cholestatic), Eosinophilic enteritis, Hemophagocytic lymphohistiocytosis, Medulloblastoma, End stage renal disease, Atopic dermatitis, Recurrent aphthous stomatitis, Depressive disorder

### Diagnostic yields

Pathogenic variants were identified in 16 out of 93 patients (17.2%); among these, 12 patients (12.9%) were diagnosed with IBMFS based on these pathogenic variants. Among the 12 patients with confirmed pathogenic variants, in 58.3% (7/12), the NGS-based target gene sequencing was used as the first-line diagnostic tool (Table 1).

The final diagnosis was modified in 12 patients with confirmed pathogenic variants, significantly influencing the treatment direction. In particular, these findings impacted the advanced treatment approach, including decisions on HSCT for some patients. For instance, one patient (patient 2) who did not recover bone marrow after intensive chemotherapy for AML was also diagnosed with FA, leading to a reduction in the intensity of the conditioning regimen for HSCT. Additionally, for patients being monitored for NSAA, the genetic analysis enabled more timely and decisive decisions on HSCT based on their medical condition (Table 2). Regarding the specific indications for the target gene sequencing use, it was implemented in 28 patients with specific medical history, 22 with persistent cytopenia lasting more than 6 months or unexplained recurrent neutropenic fever leading to 2 or more hospitalizations, 11 with changes in the pattern of cytopenia, 10 with atypical bone marrow findings requiring differential diagnosis of IBMFS, and 22 for other reasons (See Supplemental Data Table S2). Among these, pathogenic variants were identified in 9 out of 28 patients with specific medical history (32.1%), 3 out of 22 patients with persistent cytopenia or recurrent neutropenic fever (13.6%), 4 out of 11 patients with changes in the pattern of cytopenia (36.4%), 0 of 10 patients with atypical bone marrow findings requiring differential diagnosis of IBMFS (0%), and 0 of 22 patients for other reasons (0%) (Fig. 1). In this study, none of the patients who underwent NGS for recurrent infections with accompanying neutropenia exhibited clinical features suggestive of primary immunodeficiency (PID); therefore, PID screening was not performed. Patients over 12 years old at the time of NGS-based target gene sequencing showed a trend towards higher diagnostic yields, though the difference was not statistically significant (6.1% vs. 20.5%, *P* = 0.061).

**Table 2:** Diagnostic and therapeutic adjustments in patients with confirmed pathogenic variants

Pts	Sex	Age (yr)	Physical anomaly	Diagnosis before confirmed pathogenic variants	Changed diagnosis after confirmed pathogenic variants	Cytogenetic findings	Gene (Variant classification)	Zygosity	The contents of treatment or approach changes due to change in diagnosis	Outcome
1	F	8.3	-	Non-severe aplastic anemia	Diamond-Blackfan anemia	-	*RPS19* (c.156G > A, p.Trp52*, P)	Heterozygous	More promptly & definitely decision on HSCT according to patient’s medical condition	HSCT and alive
2	M	11.6	-	Acute myeloid leukemia, M5b	Acute myeloid leukemia, M5b with Fanconi anemia	46,XY, del(6)(p22), del(13)(q12q14)[20]	*FANCG* (c.1066 C > T, p.Gln356*, P)	Homozygous	Intensity reduction in conditioning regimen for HSCT	HSCT and alive
3	M	15.5	-	Recurrent aphthous stomatitis with suspected dyskeratosis congenita	Dyskeratosis congenita	-	*DKC1* (c.1058 C > T, p.Ala353 Val, P)	Hemizygous	Changes in the follow-up and further evaluation plan for concurrent malignancy	Alive (no HSCT to date)
4	F	8.2	-	Idiopathic bicytopenia with hypereosinophilia	Myeloid neoplasms with germline GATA2 mutation	47,XX, + 1, der(1;7)(q10;p10), + 8[17]/ 48,idem, + 8[4]	*GATA2* (c.1186 C > T, p.Arg396 Trp, LP)	Heterozygous	More promptly & definitely decision on HSCT	HSCT and alive
5	F	12.9	Lymphedema	Myelodysplastic syndrome	Primary lymphedema with myelodysplasia (Emberger syndrome)	45,XX,−7[18]/ 46,XX[2]; loss of 7	*GATA2* (c.1143 + 1G > A, P)	Heterozygous	Changes in the follow-up and further evaluation plan for concurrent disease (etc. sensorineural deafness, low CD4/CD8 T cell ratio)	Alive (no HSCT to date)
6	F	13.1	Skeletal anomaly	R/O Fanconi anemia	Fanconi anemia	-	*FANCA* (c.4168-2 A > G, P; c.2728_2731 del, p.Leu910Glyfs*10, LP)	Likely compound heterozygous	More promptly & definitely decision on HSCT according to patient’s medical condition	HSCT and alive
7	M	12.6	-	Idiopathic neutropenia	Cohen syndrome	-	*VPS13B* (c.5809_5810 delAT, p.Ile1937 Cysfs11, P; c.2047 C > T, p.Gln683, LP)	Likely compound heterozygous	G-CSF administration and surveillance for malignancy	Alive
8	F	10.5	-	Idiopathic neutropenia	Congenital neutropenia	-	*ELANE* (c.377 C > T, p.Ser126Leu, P)	Heterozygous	G-CSF administration and surveillance for malignancy	Alive
9	F	11.3	Short stature	Idiopathic neutropenia	Congenital neutropenia	-	*ELANE* (c.137 C > T, p.Ser46Phe, P)	Heterozygous	G-CSF administration and surveillance for malignancy	Alive
10	M	18	-	Idiopathic neutropenia	Congenital neutropenia	-	*ELANE* (c.669 C > A, p.Cys223*, P)	Heterozygous	G-CSF administration and surveillance for malignancy	Alive
11	M	22.7	Failure to thrive	Idiopathic neutropenia	Congenital neutropenia	-	*G6PC3* (c.215 delA, p.Lys72Serfs*45, P)	Homozygous	G-CSF administration and surveillance for malignancy	Alive
12	F	0.2	Failure to thrive	Idiopathic anemia	Shwachman-Diamond syndrome	-	*SBDS* (c.258 + 2 T > C, P; c.183_184 delinsCT, p.Lys62*, LP)	Likely compound heterozygous	More promptly decision on HSCT in case of changes in cytopenia pattern	Alive (no HSCT to date)

*P* Pathogenic, *LP* Likely pathogenic, *HSCT* hematopoietic stem cell transplantation, *G-CSF* granulocyte-colony stimulating factor

### Clinical characteristics of patients with confirmed pathogenic variants

The distribution of clinical features, including abnormal bone marrow findings, congenital anomalies, and medical history, did not differ significantly between the groups with and without confirmed pathogenic variants. However, the group with confirmed pathogenic variants exhibited significantly higher rates of cytogenetic abnormalities (33.3% vs. 5.4%, *P* = 0.031; not significant after multiple testing correction). Monosomy 7 was identified in one of our patients who exhibited features of MDS and lymphedema, with a confirmed *GATA2* mutation. This presentation is consistent with Emberger syndrome—an autosomal dominant disorder associated with a predisposition to AML and typically characterized by syndromic features such as congenital sensorineural deafness, lymphedema, and immunologic abnormalities [32, 33]. Der(1;7)(q10;p10), an unbalanced whole-arm translocation between chromosomes 1 and 7, is also seen in patients with myeloid neoplasms carrying germline GATA2 mutations and presenting with MDS features. Del(6)(p22), a deletion on chromosome 6p, and del(13)(q12q14), a deletion on chromosome 13q, were identified in a single patient diagnosed with AML subtype M5b and FA, who failed to recover bone marrow function following intensive chemotherapy. (Table 3).

**Table 3:** Clinical characteristics between group with confirmed pathogenic variants and group without confirmed pathogenic variants

Characteristics	Group without confirmed pathogenic variants (*N* = 81)	Group with confirmed pathogenic variants (*N* = 12)	P-value^a^
Age (years)	9.1 (0.3–31.4)	12.1 (0.2–22.7)	0.1204
Male sex	50 (61.7)	5 (41.7)	0.3150
Hemoglobin (g/dL)	10.5 (6.1–17.7)	11.1 (7.8–14.8)	0.1841
White blood cell (/μl)	3070 (90–10850)	3695 (400–7090)	0.5966
Platelet (× 10^3^/μl)	51 (10–613)	134 (22–394)	0.0991
Absolute neutrophil count (/μl)	675 (0–4394)	670.5 (0–2916)	0.6653
Lowest^b^ Hemoglobin (g/dL)	9.1 (4.5–16.7)	9.1 (5.2–12.6)	0.8626
Lowest^b^ White blood cell (/μl)	2570 (90–11400)	3135 (1110–4180)	0.2032
Lowest^b^ Platelet (× 10^3^/μl)	32 (1–462)	105.5 (3–370)	0.0524
Lowest^b^ Absolute neutrophil count (/μl)	455 (0–3591)	293 (0–3282)	0.7140
Patients with positive antinuclear antibody	13/36 (36.1)	2/5 (40.0)	1.0000
Patients with accompanying symptoms (Infection or Bleeding)	52 (64.2)	9 (66.6)	1.0000
Patients with abnormal bone marrow findings	44/56 (78.6)	6/9 (66.6)	0.4200
Patients with abnormal cytogenetics findings	3/56 (5.4)	3/9 (33.3)	0.0306
Patients with congenital anomaly	9 (11.1)	3 (25.0)	0.1834
Patients with medical history	16 (19.8)	3 (25.0)	0.7051

Values are presented as median (range) or frequency or number (%) or number/total number (%)

^a^ Continuous variables were analyzed using the t-test or Wilcoxon rank sum test, while categorical data were analyzed using the χ2 test or Fisher's exact test

^b^ Lowest was defined as the lowest value among results within 1 month before and after the gene panel

### Clinical implications of identified pathogenic variants

In all patients with modified diagnoses, the identification of pathogenic variants led to direct changes in treatment strategies and surveillance planning. These changes included adjustments in the timing or intensity of HSCT, as well as the implementation of targeted cancer screening and evaluations for syndromic features.

Specifically, a patient initially diagnosed with NSAA was found to have an *RPS19* variant, resulting in a diagnosis of DBA and a more prompt and definitive decision regarding HSCT. Another patient with AML subtype M5b harbored a *FANCG* variant, confirming FA and prompting a reduction in the intensity of the conditioning regimen for HSCT. Among patients with *GATA2* variants, one was reclassified from idiopathic bicytopenia with hypereosinophilia to myeloid neoplasms with germline GATA2 mutation, leading to earlier HSCT decision-making, while another with myelodysplastic syndrome and lymphedema was diagnosed with Emberger syndrome, prompting additional evaluations for syndromic features such as sensorineural deafness and immunologic abnormalities. A patient with recurrent aphthous stomatitis and mild leukopenia was found to have a *DKC1* variant, leading to a diagnosis of DC and implementation of malignancy surveillance. Identification of an *SBDS* variant in a patient with idiopathic anemia led to reclassification as SDS and earlier HSCT planning based on cytopenia progression. In patients initially diagnosed with idiopathic neutropenia, variants in *ELANE*, *G6PC3* or *VPS13B* supported diagnoses of congenital neutropenia or Cohen syndrome —a multisystem autosomal recessive disorder characterized by developmental delay, characteristic facial features, retinal dystrophy, and non-cyclic neutropenia that is often mild to moderate and non-fatal [34]. These findings guided the initiation of granulocyte colony-stimulating factor therapy and ongoing surveillance for hematologic malignancies (Table 2). Most of the pathogenic variants were identified in genes with well-established roles in IBMFS, including *FANCA*, *FANCG*, *RPS19*, *SBDS*, *GATA2*, *DKC1*, and *ELANE*. Less commonly observed genes, such as *G6PC3* and *VPS13B*, have also been previously associated with congenital neutropenia and syndromic disorder, including Cohen syndrome. As such, the identified variants do not represent novel findings.

### Clinical characteristics of patients with AA or MDS

There were no significant differences in clinical characteristics, proportions of patients with congenital anomalies or medical history, and rates of intervention requiring medication or HSCT between the groups of AA or MDS with and without confirmed pathogenic variants. However, the AA or MDS group with confirmed pathogenic variants exhibited significantly higher proportions of cytogenetic abnormalities (50.0% vs. 5.4%, *P* = 0.0409; not significant after multiple testing correction) (Supplemental Data Table S3).

Additionally, among patients diagnosed with AA or MDS, we compared clinical characteristics between patients presenting with a SAA pattern and those who did not. In the SAA group, no patient had confirmed pathogenic variants. However, in the NSAA group, four patients presented pathogenic variants (20.0% vs. 0%, *P* = 0.0478; not significant after multiple testing correction), and all four of these patients had accompanying abnormal cytogenetic findings (Supplemental Data Table S4).

## Discussion

In recent years, the advent of NGS strategies based on target gene sequencing has shifted IBMFS diagnosis toward a “genotyping first” approach, significantly improving clinical management and ongoing care. In our study, the diagnostic yield of the NGS-based target gene sequencing was 12.9% (12/93). In all patients with confirmed pathogenic variants, therapeutic plans, cancer surveillance, and follow-up strategies were adjusted based on the specific genetic diagnosis. In contrast, most patients without confirmed pathogenic variants underwent conventional management based on clinical features, complete blood counts, bone marrow findings, and cytogenetic analysis, including routine monitoring or standard treatment for AA or MDS. These findings reflect the high qualitative yield of NGS in guiding individualized clinical decisions and improving diagnostic accuracy in patients with suspected IBMFS.

In previous studies similar to ours, the NGS-based genomic diagnostic yield for patients suspected of IBMFS varied widely, ranging from 11.2% to 55.5% [3, 5, 18, 23–26]. One study reported that an NGS panel comprising 203 genes identified diagnoses for 65 of 130 patients with suspected IBMFS, achieving an overall molecular diagnostic rate of 50% [3]. Another study involving 204 patients used two versions of the NGS tool (version 1 included 129 genes, and version 2 involved 145 genes) and achieved an overall diagnostic rate of 44% (91/204) [5]. Additional studies have reported the following diagnostic yields: 44% (53/121) using a 184-gene panel on 121 patients [18], 38% (61/158) using a 72-gene panel on 158 patients [23], 55.5% (15/27) using a 33-gene panel on 27 patients [24], and 11.2% (8/71) using an 85-gene panel on 71 patients [25]. While other studies include congenital anemia genes in their IBMFS panels, our institution uses a separate panel for anemia. Consequently, patients with isolated anemia may not have been tested with the IBMFS panel, potentially affecting our yield.

Our study revealed that using NGS, pathogenic variants were significantly more frequently identified in patients with cytogenetic abnormalities compared to those with a normal karyotype. Monosomy 7, detected in our patient, is well-known as a frequently concurrent cytogenetic abnormality in patients with GATA2-related MDS [32]. The unbalanced whole-arm chromosomal translocation Der(1;7)(q10;p10), which results in trisomy 1q and deletion of 7q, has been reported in multiple pediatric patients with primary MDS. However, in patients with GATA2-related MDS, it has only been documented in a single report [33]. There are no reported cases of del(6)(p22) or del(13)(q12q14) in patients with AML or FA. The association between IBMFS and cytogenetic abnormalities is well-established, and cytogenetic analysis is recommended for all suspected IBMFS patients. For GATA2 deficiency, SAMD9/SAMD9L syndrome, SDS, and DC, which are frequently associated with cytogenetic abnormalities, regular cytogenetic evaluations are advised every 12–18 months [35]. Notably, genetic abnormalities were more common in patients with NSAA than in those with SAA. Consistent with this finding, a retrospective cohort study of 325 children and adults found that patients with constitutional BMF more frequently present with NSAA than with SAA or VSAA [36]. A previous study involving 106 pediatric and adult suspected IBMFS patients reported a higher diagnostic yield (40.5% vs. 55.5%, P-value not provided) using a 33-gene NGS panel in patients aged 12 years or older [26]. Similarly, our cohort — which primarily consisted of pediatric patients — showed a trend toward higher diagnostic yields in patients aged 12 years or older, although this was not statistically significant. This finding may imply that cytopenias occurring in younger children are more likely to be attributable to non-genetic causes. Patients with specific medical histories or changes in the pattern of cytopenia (progression from single lineage to multilineage, aggravation with increased transfusion needs or prolonged aplasia during chemotherapy for malignancies) showed higher yields of 32.1% and 36.4%, respectively, compared to the overall yield. Thus, genetic diagnosis of IBMFS via NGS is particularly promising in patients with cytogenetic abnormalities, NSAA, specific medical history, or changes in the pattern of cytopenia.

In this study, patients initially diagnosed with AA or MDS were grouped together, as most of the nine patients with MDS were hypoplastic MDS (hMDS), characterized by bone marrow hypocellularity and peripheral blood cytopenia resembling AA. Moreover, hMDS overlaps with normo-/hypercellular MDS (NH-MDS), which is associated with dyspoiesis, chromosomal abnormalities, and a higher AML risk. Recurrent genetic and epigenetic alterations occur in hMDS, NH-MDS, and AA at varying frequencies, with no significant differences [2, 37–39].

Four patients were identified as genetic carriers despite having pathogenic variants (See Supplemental Data Table S5). Among them, a pediatric patient diagnosed with B-lymphoblastic leukemia was found to be a *FANCA* carrier. After consolidation chemotherapy, the patient remained in complete remission; however, bone marrow suppression persisted, necessitating ongoing transfusion support. Consequently, HSCT was performed due to aplasia. Despite identifying a genetic abnormality in one gene, a definitive diagnosis was not established, suggesting that comprehensive sequencing, such as WGS, considering VUS and undetected regions including deep introns, may be necessary in the future. This case underscores the necessity of genetic screening for IBMFS among patients with hematologic malignancies and complications like prolonged aplasia during treatment.

The understanding of known pathogenic variants can evolve over time. Some FA genes, such as *FANCD1* [*BRCA2*] and *FANCJ* [*BRIP1*], act as cancer predisposition genes in a monoallelic autosomal dominant manner [40, 41]. After genetic diagnosis of DBA, cancer screening and surveillance for colorectal cancer and osteogenic sarcoma are also emphasized [42]. Recent reports highlight emerging germline variants linked to HPSC failure pathogenesis [43]. Disorders such as GATA2 deficiency, involving transcription defects, and SAMD9/SAMD9L syndrome, related to growth regulation, are classified within IBMFS. Germline heterozygous mutations in *GATA2* present with a spectrum of phenotypes, from mild cytopenia to severe immunodeficiency, and often progress to advanced myeloid neoplasia and life-threatening infections, requiring timely stem cell transplantation [36]. Gain-of-function mutations in *SAMD9* and *SAMD9L*, located on chromosome 7, enhance antiproliferative effects, leading to pancytopenia and restricted growth or organ hypoplasia in non-hematopoietic tissues [44]. Family studies involving co-occurring mutations in *FANCC* and *CHEK2* underscore the importance of analyzing not only primary phenotype-determining genes but also those that can significantly influence the phenotype when occurring together [45]. During the study, we identified patients where IBMFS were strongly suspected, despite NGS-based target gene sequencing did not detect any relevant findings. Limitations in genetic diagnostic tools, including difficulties in determining the pathogenicity of rare variants and gaps in the current understanding of IBMFS, may have contributed to these findings. In previous studies, the limitations of the target gene sequencing were overcome through additional analyses such as WES, WGS, and chromosomal microarray [3, 5]. However, one clear limitation of our study is the inability to incorporate these additional methods. While each molecular approach has its merits, they cannot fully replace one another. WES, although effective for gene discovery, lacks complete coverage of all clinically relevant exons. In contrast, target gene capture provides high-depth coverage across all genes of interest. Therefore, a preferable strategy to enhance genetic diagnostic yield could involve initial screening with an updated panel, followed by selecting patients for WES or even WGS based on clinical suspicion [5, 8, 25]. NGS-based target gene sequencing typically costs approximately USD 850 per test. Although the overall diagnostic yield of 12.9% in our study is not higher than that reported in some previous studies, it may still be considered cost-effective for pediatric and AYA patients with cytopenia who meet the proposed testing criteria. The cost-effectiveness is likely greater in specific subgroups with higher diagnostic yield trends—such as those with specific medical history, changing in patterns of cytopenia, cytogenetic abnormalities, or NSAA. In these patients, prioritizing NGS-based targeted gene sequencing may be particularly beneficial, even in resource-limited settings. These findings highlight the need for larger, multicenter studies to validate our results and establish standardized guidelines to enhance the diagnostic yield of NGS in IBMFS.

Based on previous and forthcoming reports, the diagnostic yield can be further enhanced by including additional genes for diagnosing both classified and unclassified IBMFS. Unclassified IBMFS encompasses a diverse set of genetic disorders, including newly identified syndromes and atypical variations of recognized IBMFS. To enhance the diagnostic process for classified IBMFS, specific genes can be considered: *TERC* for DC, *RPS17* for DBA, *CYCS*, *GP1BA*, *ITGA2B*, *ITGB3*, *RBM8 A*, and *TUBB1* for hereditary thrombocytopenia, as well as *ABCB7*, *PUS1*, *ALAS2*, and *SLC25 A38* for congenital anemia. In unclassified IBMFS, relevant genes include *LIG4* (LIG4 syndrome: pancytopenia, severe immunodeficiency), *ALDH2*/*ADH5* (AMeD syndrome: AA, intellectual disability, dwarfism), *RECQL4* (Rothmund-Thomson syndrome: bilineage cytopenia), *AP3B1* (Hermansky-Pudlak syndrome type 2: bone marrow failure), *SRP72* (familial aplasia and myelodysplasia), and *MYH9* (MYH9-related disorder: thrombocytopenia) [3, 5, 18, 23–25].

This study has several limitations. First, the retrospective design introduces inherent risks of selection bias and unmeasured confounding, particularly due to incomplete or heterogeneous clinical data. The relatively small sample size and the potential for referral bias may also limit the generalizability of our findings to broader patient populations. Additionally, while germline status was inferred from DNA extracted from peripheral blood or bone marrow, non-hematopoietic tissue such as buccal mucosa was not consistently available. This may reduce the ability to definitively distinguish between somatic and germline variants, particularly in patients with hematologic malignancies or clonal hematopoiesis. Furthermore, although autosomal recessive inheritance was suspected in patients with biallelic pathogenic variants, parental or familial testing was not performed in all patients. In compound heterozygous patients, the possibility of de novo mutations cannot be completely excluded without segregation data. Finally, as this study was cross-sectional in nature, it does not provide insight into the long-term clinical impact of genetic diagnoses. Future prospective and longitudinal studies will be essential to assess how genetic findings influence patient outcomes, including survival, quality of life, treatment decisions, and the risk of secondary malignancies.

As the use of NGS expands, it is equally important to address the ethical implications of broader genetic testing. The ACMG recommends actively reporting clinically actionable incidental or secondary findings—those unrelated to the primary indication—when identified through exome or genome sequencing. To ensure responsible disclosure, pre- and post-test genetic counseling is essential, helping patients and families understand the potential outcomes of testing, including unexpected results [46]. These ethical considerations are particularly relevant in pediatric settings, where decisions may have long-term implications. Notably, no incidental or secondary findings were identified in our study cohort.

In conclusion, this study demonstrated the diagnostic value of NGS-based target gene sequencing for pediatric and AYA patients suspected of genetic hematologic disorders, in conjunction with traditional bone marrow examinations, chromosomal analyses, and chromosomal breakage tests. Importantly, the diagnostic yield was higher in specific subgroups where NGS appears to be particularly effective, such as patients with cytogenetic abnormalities, NSAA, a specific medical history and changes in patterns of cytopenia, including prolonged aplasia during chemotherapy for malignancies. These findings support the expansion of clinical indications for NGS to include these high-yield groups and underscore the need for multicenter studies and the development of standardized guidelines to optimize its use in IBMFS.

## Supplementary Information

Below is the link to the electronic supplementary material.

ESM 1 (DOCX 32.8 KB)

## Data Availability

No datasets were generated or analysed during the current study.
