# Supplementary material for: Clinical usefulness of next-generation sequencing-based target gene sequencing in diagnosis of inherited bone marrow failure syndrome
Source: Ann Hematol. 2025 May 13;104(5):2693–706. doi: 10.1007/s00277-025-06392-0 (PMC12141389; doi:10.1007/s00277-025-06392-0)
Supplement: Supplementary file 1 — (DOCX 32.8 KB) [file 277_2025_6392_MOESM1_ESM.docx]

| **Supplemental Table 1.** IBMFS Genes Included in NGS-Based Target Gene Panels | | | | | | | | |
| --- | --- | --- | --- | --- | --- | --- | --- | --- |
| FA | DC | DBA | Congenital neutropenia | Hereditary thrombocytopenia | SDS | Congenital anemia | Other IBMFS | AML/MDS |
| *FANCA* | *CTC1* | *GATA1* | *ELANE* | *ANKRD26* | *SBDS* | *CDAN1* | *GATA2* | *TP53* |
| *FANCB* | *DKC1* | *RPS7* | *G6PC3* | *MPL* | *EFL1* |  | *SAMD9* | *CEBPA* |
| *FANCC* | *NHP2* | *RPS10* | *GFI1* |  |  |  | *SAMD9L* | *RUNX1* |
| *BRCA2 (FANCD1)* | *NOP10* | *RPS19* | *HAX1* |  |  |  | *NBN* | *DDX41* |
| *FANCD2* | *RTEL1* | *RPS24* | *WAS* |  |  |  | *PMS2* | *ETV6* |
| *FANCE* | *TERT* | *RPS26* | *JAGN1* |  |  |  | *NF1* | *PAX5* |
| *FANCF* | *TINF2* | *RPL5* | *TCIRG1* |  |  |  | *VPS13B* | *PTPN11* |
| *FANCG* | *WRAP53* | *RPL11* | *CLPB* |  |  |  | *EPCAM* |  |
| *FANCI* | *ACD* | *RPL35A* | *CSF3R* |  |  |  | *BLM* |  |
| *BRIP1*  *(FANCJ)* | *PARN* | *RPL18,* |  |  |  |  | *POT1* |  |
| *FANCL* |  | *RPL26* | *VPS45* |  |  |  | *ATM* |  |
| *FANCM* |  | *RPS29* | *SRP54* |  |  |  | *MLH1* |  |
| *PALB2 (FANCN)* |  | *RPL15,* |  |  |  |  | *MSH2* |  |
| *RAD51C (FANCO)* |  | *RPL27* |  |  |  |  | *MSH6* |  |
| *SLX4 (FANCP)* |  | *RPL35,* |  |  |  |  | *SLC37A4* |  |
| *ERCC4 (FANCQ)* |  | *RPS15A,* |  |  |  |  | *TAZ* |  |
| *RAD51*  *(FANCR)* |  | *RPS28,* |  |  |  |  | *DNAJC21* |  |
| *BRCA1 (FANCS)* |  | *TSR2* |  |  |  |  | *CXCR4* |  |
| *UBE2T (FANCT)* |  |  |  |  |  |  | *SH2B3* |  |
| *XRCC2*  *(FANCU)* |  |  |  |  |  |  | *STK4* |  |
| *MAD2L2 (FANCV)* |  |  |  |  |  |  | *STN1* |  |
| *RFWD3 (FANCW)* |  |  |  |  |  |  | *CREBBP* |  |

FA=Fanconi anemia, DC=Dyskeratosis congenita, DBA=Diamond-Blackfan anemia, SDS=Shwachman-Diamond syndrome, IBMFS=Inherited bone marrow failure syndromes, AML/MDS=Acute myeloid leukemia/Myelodysplastic syndromes.

**Supplemental Table 2.** Detailed Information on 'Other reasons' for target gene sequencing

| Reason for testing | Description of circumstances | Values |
| --- | --- | --- |
| Late onset with mild cytopenia | Did not meet indications for target gene sequencing, but tested due to atypical onset at age over 12. | n=7 |
| Refractory to treatment | Diagnosed with SAA and treated with IST, but showed no clinical response. | n=1 |
| Parental request | Solely at the parents’ request, without clear clinical indications. | n=2 |
|  | Solely at the parents’ request, in patients intermittently meeting SAA criteria but not clearly indicated for HSCT or IST. | n=12 |

SAA=severe aplastic anemia, HSCT= hematopoietic stem cell transplantation, IST= immunosuppressive therapy

| **Supplemental Table 3.** Clinical characteristics and course between group of AA or MDS without confirmed pathogenic variants and group of AA or MDS with confirmed pathogenic variants | | | |
| --- | --- | --- | --- |
| Characteristics | Group of AA or MDS without confirmed pathogenic variants  (N = 37) | Group of AA or MDS with confirmed pathogenic variants  (N = 4) | P-value^a^ |
| Age (years) | 10.0 (1.1–31.4) | 10.6 (8.2–13.1) | 0.8951 |
| Male sex | 19 (51.4) | 0 (0) | 0.1531 |
| Hemoglobin (g/dL) | 8.7 (6.1-11.9) | 9.75 (8.1-10.9) | 0.3470 |
| White blood cell (/μl) | 2395 (90-7890) | 3245 (2280-3880) | 0.1533 |
| Platelet (×10^3^/μl) | 32.5 (10-161) | 65 (22-368) | 0.1293 |
| Absolute neutrophil count (/μl) | 580.5 (0-2614) | 1160 (745-1541) | 0.0187 |
| Lowest^†^ Hemoglobin (g/dL) | 7.2 (4.5-11.2) | 6.2 (5.2-8.1) | 0.2541 |
| Lowest^†^ White blood cell (/μl) | 2085 (90-5100) | 2875 (1110-3280) | 0.3282 |
| Lowest^†^ Platelet (×10^3^/μl) | 15.5 (1-161) | 37 (3-251) | 0.1406 |
| Lowest^†^ Absolute neutrophil count (/μl) | 435.5 (0-1836) | 725.5 (211-897) | 0.1663 |
| Patients with congenital anomaly | 5 (13.5) | 1 (25.0) | 0.4830 |
| Patients with medical history | 8 (21.6) | 1 (25.0) | 1.0000 |
| Patients with abnormal cytogenetics findings | 2 (5.4) | 2 (50.0) | 0.0409 |
| Patients underwent observation | 11 (29.7) | 0 (0) | 0.5590 |
| Patients underwent medication | 2 (5.4) | 0 (0) | 1.0000 |
| Patients underwent HSCT | 24 (64.9) | 4 (100) | 0.2883 |

Values are presented as median (range) or frequency or number (%)

HSCT = hematopoietic stem cell transplantation, AA = aplastic anemia, MDS = myelodysplastic syndromes.

^a^ Continuous variables were analyzed using the t-test or Wilcoxon rank sum test, while categorical data were analyzed using the χ2 test or Fisher's exact test.

^b^ Lowest was defined as the lowest value among results within one month before and after the Gene panel.

| **Supplemental Table 4.** Clinical characteristics between group with non-severe AA and group with severe AA | | | |
| --- | --- | --- | --- |
| Characteristics | Group  with non-severe AA  (N = 20) | Group  with severe AA  (N = 21) | P-value^a^ |
| Age (years) | 13.0 (4.8–31.4) | 8.0 (1.1–24.1) | 0.0067 |
| Male sex | 10 (50) | 9 (42.9) | 0.8846 |
| Hemoglobin (g/dL) | 8.85 (6.1-11.9) | 8.95 (6.8-10.7) | 0.7558 |
| White blood cell (/μl) | 2780 (1140-5900) | 1480 (90-7890) | 0.0189 |
| Platelet (×10^3^/μl) | 35 (10-368) | 35 (10-92) | 0.6762 |
| Absolute neutrophil count (/μl) | 797 (103-2614) | 334.5 (0-957) | <0.001 |
| Lowest^b^ Hemoglobin (g/dL) | 7.05 (5.2-11.2) | 7.2 (4.5-9.9) | 0.6402 |
| Lowest^b^ White blood cell (/μl) | 2265 (1110-5100) | 1325 (90-3600) | 0.0237 |
| Lowest^b^ Platelet (×10^3^/ μl) | 18.5 (3-251) | 12.0 (1-38) | 0.0131 |
| Lowest^b^ Absolute neutrophil count (/μl) | 657.5 (80-1836) | 223.5 (0-741) | <0.001 |
| Patients with congenital anomaly | 3 (15.0) | 3 (14.3) | 1.0000 |
| Patients with medical history | 3 (15.0) | 6 (28.6) | 0.4537 |
| Patients with abnormal cytogenetics findings | 4 (20.0) | 0 (0) | 0.0478 |
| Patients with confirmed pathogenic variants | 4 (20.0) | 0 (0) | 0.0478 |

Values are presented as median (range) or frequency or number (%)

AA = aplastic anemia.

^a^ Continuous variables were analyzed using the t-test or Wilcoxon rank sum test, while categorical data were analyzed using the χ2 test or Fisher's exact test.

^b^ Lowest was defined as the lowest value among results within one month before and after the gene panel use.

| **Supplemental Table 5.** Summary of Genetic Carriers (N = 4) | | | | | |
| --- | --- | --- | --- | --- | --- |
| Patient | Sex | Age(yr) | Diagnosis | Type of genetic carrier | Gene variant |
| 1 | M | 10.9 | Idiopathic neutropenia | Genetic carrier of Fanconi anemia | *FANCA* |
| 2 | M | 10.8 | Idiopathic neutropenia | Genetic carrier of Fanconi anemia | *BRCA2* |
| 3 | M | 8.2 | B-lymphoblastic Leukemia | Genetic carrier of Fanconi anemia | *FANCA* |
| 4 | M | 1 | Idiopathic neutropenia | Genetic carrier of Mismatch repair cancer syndrome 4 | *PMS2* |
